# Development of Microencapsulation Delivery System for Long-Term Preservation of Probiotics as Biotherapeutics Agent

**DOI:** 10.1155/2013/620719

**Published:** 2013-08-21

**Authors:** Himanshu K. Solanki, Dipak D. Pawar, Dushyant A. Shah, Vipul D. Prajapati, Girish K. Jani, Akil M. Mulla, Prachi M. Thakar

**Affiliations:** ^1^Department of Pharmaceutics, S.S.R. College of Pharmacy, Sayli-Silvassa Road, Sayli, Silvassa, Dora and Nagar Haveli 396230, India; ^2^Hemchandracharya North Gujarat University, Patan, Gujarat 384265, India; ^3^APMC College of Pharmaceutical Education and Research, Motipura, Himmatnagar, Sabarkantha 383001, India

## Abstract

The administration of probiotic bacteria for health benefit has rapidly expanded in recent years, with a global market worth $32.6 billion predicted by 2014. The oral administration of most of the probiotics results in the lack of ability to survive in a high proportion of the harsh conditions of acidity and bile concentration commonly encountered in the gastrointestinal tract of humans. Providing probiotic living cells with a physical barrier against adverse environmental conditions is therefore an approach currently receiving considerable interest. Probiotic encapsulation technology has the potential to protect microorganisms and to deliver them into the gut. However, there are still many challenges to overcome with respect to the microencapsulation process and the conditions prevailing in the gut. This review focuses mainly on the methodological approach of probiotic encapsulation including biomaterials selection and choice of appropriate technology in detailed manner.

## 1. Introduction

As described by the Food and Agriculture Association of the United Nations (FAO) and World Health Organization (WHO), probiotic are a group of live microorganisms that, when administered in adequate amounts, confer a health benefit on the host [1]. Probiotic is a term that means “for life” and defined as “live microorganisms that beneficially affect the host's health by improving its microbial balance” [2]. *Lactobacillus *and *Bifidobacteria* are the two most common types of microbes which are extensively used as probiotics [2, 3]. The use of probiotic bacterial culture stimulates the growth of preferred microorganisms, crowds out potentially harmful bacteria, and reinforces the body's natural defense mechanisms [4]. Some bacterial strains that have been widely discussed in the literature are outlined in Table 1 along with their therapeutic uses. 

Lifestyle and eating habits play an important role in the overall health of individuals. Recently the use of probiotics for health benefits has increased, and hence it has created a huge market worldwide [5]. In the development of effective and safe encapsulated product, it is essential to maintain the adequate number of viable cells during the shelf life of the product as well as during the gastrointestinal (GI) tract transit after consumption [6–10]. 

Normally, any probiotic product must contain at least 10^6^–10^7^ cfu of viable probiotic bacteria per g of the product at the time of its consumption to exert beneficial effects on human health [1]. To overcome difficulty during development, microencapsulation technique is utilized to increase the viabilitys; of probiotic also several studies are carried out to investigate their role in different conditions in probiotic exposed [11–18].

### 1.1. Purpose of Microencapsulation

The purpose of microencapsulation of probiotic is to protects certain compound or biological cells against surrounding environment which destruct the core. It protects the bacteria from heat, oxygen, and moisture and also improves the flow properties during formulation development. It can be used for different drug delivery system and nowadays to apply for the encapsulation of probiotics in food product [19–21].

**Figure 1:**
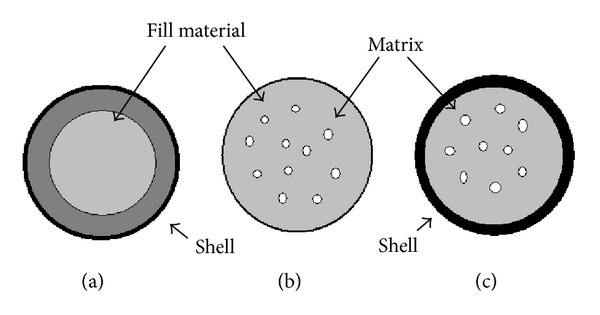
Schematic representation of encapsulation systems: (a) reservoir type, (b) matrix type, and (c) coated matrix type.

The core material is encapsulated in the food grade matrix type coating material. In the food industry, these materials form a barrier to protect the core material against the GI environment using different encapsulation systems as shown in Figure 1 [21]. 

Finally, microencapsulation gives structure and innovative system to the core material for the probiotic food product. Physicochemical properties of coating material affect the viability of encapsulated probiotic cells. Type and concentration of coating material, particle size, initial cell number, and bacterial strains are important during formulation [22].

### 1.2. Structure of Microcapsule

**Figure 2:**
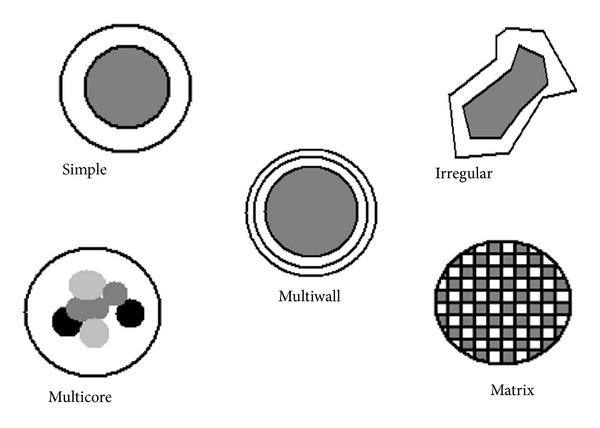
Various forms of microcapsule used in the food industry [19].

Microcapsules, formed by using natural materials like sugar, gums, protein, lipid, and synthetic or modified polymers, can be formulated as gel beads or in dried powder form. The formed smooth or irregular microcapsules lack their encapsulation efficiency because of the presence of pores [23]. The coating material is classified on the basis of the matrix material such as with a single wall material like sodium alginate, or a mixture material such as xanthan, gellan gum, alginate, and Chitosan. Coating material also affects the structure of microcapsule. Generally sodium alginate produces microcapsules with smooth surface [24], while slow gelling property of milk results in formation of irregular shaped capsule [25, 26]. Different shapes of microspheres are shown in Figure 2.

### 1.3. Advantages of Microencapsulated Probiotics

- It protects and enhances survival of bacteria in foods.
- It allows entrapped probiotic microorganisms to be incorporated into dairy products such as yogurt, cheese, and frozen milk product.
- About 40% of *Lactobacilli *survive in frozen ice cream when entrapped in calcium alginate sphere than free cells [27].
- The encapsulation of *Bifidobacteria *significantly improves survival, compared to free cells, throughout storage from 43%–44% to 50%–60% in frozen dairy product [28].
- Microencapsulated form of *B*. *pseudolongum* exhibits improvement of survival in a simulated gastric environment when compared to free viable microorganisms [29, 30].

## 2. Factor Affecting Microencapsulation Effectiveness of Probiotics

For evaluation of effectiveness of probiotic encapsulation process different parameters are considered such as viability maintenance after encountering detrimental environmental conditions, cell release/recovery ability, and hardening time (time needed for capsule formation). Different factors affecting the microencapsulation are discussed below [23, 31].

### 2.1. Effect of Various Biomaterials on Viability of Probiotics

A wide variety of biomaterials have been used by researchers in order to check their effects on the process of microencapsulation as well as on the viability of probiotic bacteria. Supported report is shown in Table 2.

### 2.2. Capsule Characteristics with Respect to the Surrounding Environment

Selection of capsular material with respect to the surrounding environment is very important. When the microcapsule is formed using alginate and different combination, leaks the calcium ions from alginate capsule structure leading to its decomposition. Hence it should be avoided from the highly acidic environment. If probiotic cells are to be targeted in the small intestine, then selection of capsule material(s) should be such that their decomposition occurs after subjecting them to the small intestine pH or pancreatic enzymes. If the beads are to be retained in the large intestine, it is preferable to be tolerant against the pancreas and small intestine conditions. However, this is not always easily achievable due to the restrictions in the chemical characteristics of encapsulation materials. Generally, all the capsules must be resistant to the acidic conditions of gastric juices [32]. Sometimes it is necessary to use special types of hydrophobic components of encapsulation to make the beads tolerant against moisture.

### 2.3. Coating of the Capsule

Efficient coating of capsule improves its physicochemical property. For example, shell coating on the alginate capsules makes them resistant to the chelating agents of calcium ions and also increases their mechanical strength.

### 2.4. Concentration of Capsule Making Solution and Bead Diameter

Concentration of capsule making solution and final bead diameter are factors which affect encapsulation efficiency. As bead diameter increases, it causes inappropriate mouth feel and flavor. Furthermore, increasing capsule diameter decreases digestibility by pancreatic enzyme. 

### 2.5. Environmental Conditions

Physiology of the GI tract is important during the probiotic encapsulation process (Table 3) [205]. 

Environmental factors are also found to reduce encapsulation effectiveness.

### 2.6. Modification of Capsule Materials

Chemical modification of capsular material improves encapsulation effectiveness. Structural modification of the capsule materials is by direct structural changes and/or addition of special additives. 

### 2.7. Initial Concentration of Microbial Cells

As concentration of microbial cells in the encapsulation solution increases, the number of entrapped cells in each bead (cell load) increases and, as a result, quantitative efficiency of encapsulation increases. If cell load exceeds the limit, softening of capsule structure occurs.

### 2.8. Conditions of Processing Factors

Microencapsulation processes such as freeze drying, spray drying, micronization, and storage conditions are employed in order to avoid injuries to the beads and contained cells. 

## 3. Formulation Technology for Microencapsulation of Probiotics

The presence of diverse condition in human digestive system makes designing of the probiotic release system difficult. Hence, highly tailored system like specific target location system is required [205].

**Figure 3:**
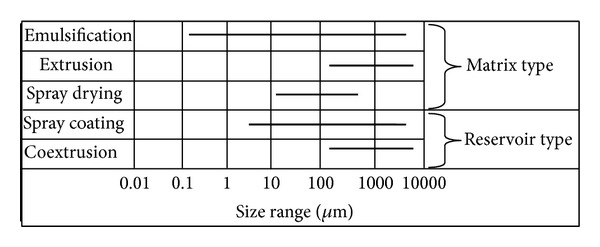
Probiotic encapsulation technologies: size range provided by each technique.

Probiotic cell is commonly encapsulated by extrusion, emulsion, and spray drying. In these methods, probiotic bacteria are entrapped in the gel matrix using different gel forming mechanisms [206]. Whereas probiotic are living cells, the condition for implementation technology are designed to maintain cell viability, and solvents involved in the encapsulation technology must be nontoxic [207]. In Figure 3, it the different types of particles obtained (matrix or reservoir type) by each method can be seen Figure 3. 

The ability of microorganisms to survive and multiply in the host strongly influences their probiotic benefits. 

Microencapsulation techniques are divided into two parts:

- encapsulation process,
- drying process.

### 3.1. Encapsulation Process

There are two basic techniques of microencapsulation that are used for encapsulation of probiotic bacteria. These encapsulated probiotics are then used for biomass production and also in various food products as functional food ingredients. 

Depending on the method used, the two methods are extruded or droplet method and emulsion or two phase system method. From various studies it has been concluded that encapsulation by both of these methods has increased the viability of probiotic bacteria more than 80%.

#### 3.1.1. Extrusion Technique for Microencapsulation

**Figure 4:**
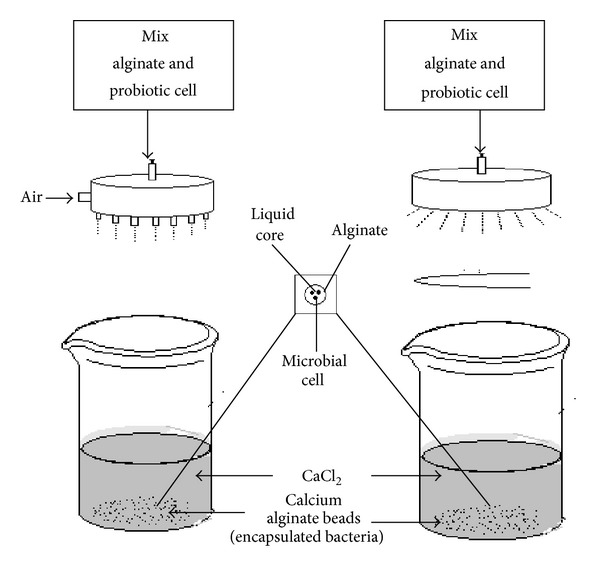
Extrusion technologies: simple needle droplet generator that usually is air driven (a) and pinning disk (b). The probiotic cells are added to the hydrocolloid solution and dripped through a syringe needle or a nozzle spray machine in the form of droplets which are allowed to free fall into a hardening solution such as calcium chloride.

It is the oldest common technique for probiotic formulation [208]. Extrusion method in the case of alginate capsule consists of the following stages: preparation of hydrocolloid solution and the addition of probiotic cell in hydrocolloid solution to form cell suspension. These cells suspension is passed through the syringe needle to form droplets which are directly dripped into the hardening solution containimg cations like calcium. When the droplets come in contact with hardening solution, alginate polymers surround the core to form a three-dimensional lattice structure by cross-linking calcium ions as shown in Figure 4 [22, 29, 146, 209, 210]. 

Thereby entrapping the core material separated from liquid bath and is dried using a suitable technology. Formation of gel by alginate solution (0.6%) would be possible if calcium ion (0.3 M) is present [193]. Usually, alginate is used in the range of 1-2% and 0.005–1.5 M calcium chloride concentration. Generally, the diameter of forming beads in this method (2–5 mm) is larger than those formed in the emulsion method. Bead diameter is affected by concentration and viscosity of alginate solution and distance between the syringe and hardening solution, and diameter of extruder orifice affects the size of bead [156]. Bead diameter decreases along with increasing concentration and viscosity of the encapsulation solution. Using low glucuronic alginate, formation of beads with smaller diameter is possible [211]. For production of alginate capsule with Chitosan coat, alginate solution is dripped into the hardening batch containing calcium chloride and Chitosan [201, 212]. Soaking of alginate beads in the Chitosan solution (0.1%, pH 6.5) for 20 min forms beads with good properties [12]. 

Review work on this technique for probiotic microencapsulation is listed in Tables 4 and 5.

#### 3.1.2. Emulsion Technique for Microencapsulation

It is successfully applied for the microencapsulation of lactic acid bacteria [170, 213]. In this method, small volume of cell/polymer slurry (dispersed phase) is added to the large volume of vegetable oil (as a continuous phase) such as soy oil, sunflower, corn, and light paraffin oil [174]. After the formation of emulsion, cross-linking is required to form gels. Gelification is done by different mechanisms like ionic, enzymatic, and interfacial polymerization as discussed next. Reported works on this technique are listed in Table 6.

It can be easily scaled up, and the diameter of producing beads is considerably smaller (25 *μ*m–2 mm). It is costly due to need of vegetable oil, surfactant, and emulsifier (Tween80 (0.2%)) for encapsulation in an emulsion [27, 175]. 

#### 3.1.3. Emulsification Ionic Gelification

**Figure 5:**
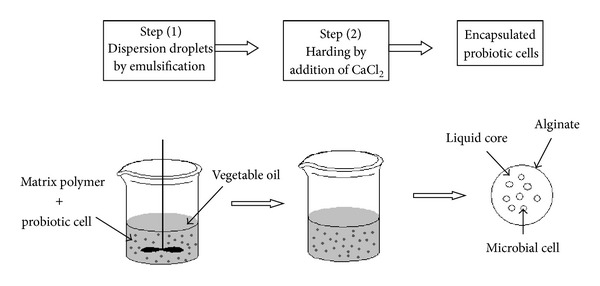
Schematic presentation of emulsification procedure: a small volume of the cell polymer suspension (i.e., the discontinuous phase) is added to a large volume of vegetable oil (i.e., the continuous phase). The mixture is then homogenized to form a water-in-oil emulsion. Once the water-in-oil emulsion is formed, the water-soluble polymer must be insolubilized to form tiny gel particles within the oil phase.

Emulsification is a chemical technique to encapsulate probiotic using alginate, carrageenan and pectin as an encapsulating material (Figure 5).

Once W/O emulsion is formed, water soluble polymer becomes insoluble after addition of ions of calcium chloride, by means of cross-linking forming gel particles in the oil phase. The smallest particle of the aqueous phase in W/O phase emulsion will lead to the formation of beads with smaller diameters. Agitation rate and type of emulsifier also affects the diameter of the beads [29, 214]. Microbeads produced by this technique are recovered by membrane filtration technology [29]. 

#### 3.1.4. Emulsification and Enzymatic Gelification

**Figure 6:**
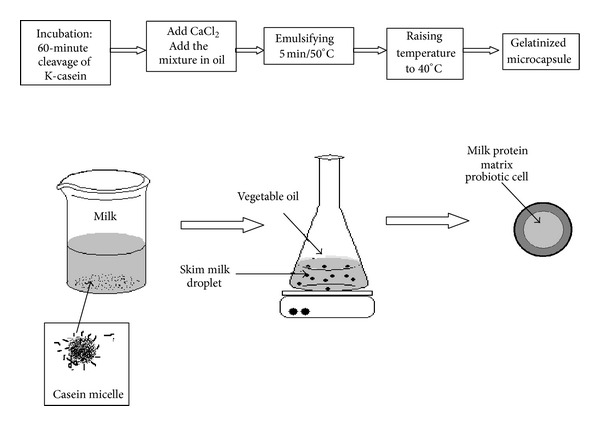
Schematic presentation of the microencapsulation of probiotic cells by means of rennet-gelation of milk proteins: The principle of the technique is based on using dairy proteins which have been put in contact with rennet at low temperature. This allows keeping a liquid system where *κ*-casein is cleaved by the enzyme. After that, dairy proteins have been emulsified in a cold oil to form water in oil emulsion. Thermal induction of enzymatic coagulation allows proteins flocculation and provides microparticles where probiotics are dispersed in coagulated dairy proteins.

In some countries, use of coating materials such as *κ*-carrageenan, gellan gum, or xanthan is not allowed in dairy product [141]. So milk protein is used to encapsulate probiotics by means of an enzyme-induced gelation [215, 216]. Milk proteins have excellent gelation properties and are a natural vehicle for probiotics [217]. This method gives water insoluble and spherical particles [215]. This method is an example of encapsulation by means of rennet gelation as shown in Figure 6, which is based on the principle of using dairy proteins which have been put in contact with rennet at low temperature. 

This allows keeping a liquid system where *κ*-casein is cleaved by the enzyme. After that, dairy proteins are emulsified in a cold oil to form water in oil emulsion. Thermal induction of enzymatic coagulation allows protein flocculation and provides microparticles.

#### 3.1.5. Emulsification and Interfacial Polymerization

This technique is a single step. It requires formation of an emulsion in which discontinuous phase contains an aqueous suspension of the cell and continuous phase contains organic solvent. To initiate the polymerization reaction, biocompatible agent which is soluble in the continuous phase is added. The drops of probiotic cell are coated to form thin and strong membrane [194]. Productivity of microorganisms is improved by interfacial polymerization in fermentation [218]. 

### 3.2. Drying Process for Microencapsulation

Drying improves stability of the encapsulated culture during prolonged storage. But the drying process causes some injuries to the microbeads, release of some cells, and reducing the viability of cells. Spray drying, freeze drying, and fluidized bed drying are common drying technology of probiotics used in industry and are summarized next [219].

#### 3.2.1. Spray Drying

**Figure 7:**
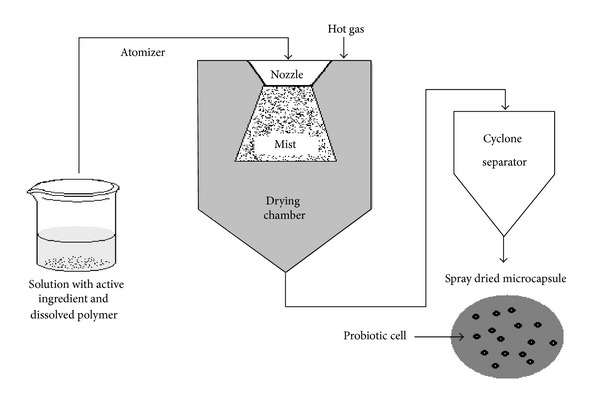
*Schematic presentation of the spray-drying procedure*: The solution is pressured and then atomized to form a “mist” into the drying chamber. The hot gas (air or nitrogen) is blown into the drying chamber too. This hot gas allows the evaporation of the solvent. The capsules are then transported to a cyclone separator for recovery.

A solution containing probiotic living cells and the dissolved polymer matrix is prepared by using gum Arabic and starches because they tend to form a spherical microparticle during the drying process (Figure 7) [22, 209, 210]. 

In drying process, probiotic cell loses viability due to physical injury to microencapsule and heat generation (Table 8) [23]. So the loss of probiotic cell can be reduced by using proper cryoprotectant during freeze drying, optimizing the inlet and outlet temperature for spray drying [206] (Table 7).

Table 7 Presents the coating materials and temperatures used in this technique for probiotic microencapsulation.

#### 3.2.2. Freeze Drying

In this technique, the solvent is frozen and removed via sublimation [220]. Freezing causes damage to the cell membrane due to ice crystal formation and also imparts stress condition by high osmolarity. It has been traditionally used to stabilize probiotic bacteria, but the combination of freeze-drying and encapsulation is relatively new concept. Recently, *Lactobacillus* F19 and *Bifidobacterium Bb12* cells were first encapsulated into enzymatically gelled sodium caseinate, and gel particles were freeze-dried to study the storage stability [17]. They reported better postdrying survival and storage viability for encapsulated cell compared to free cell. In other recent work, gelatinized starch and lecithin were incorporated into the alginate microcapsule containing probiotic organisms in encapsulated form, and beads were freeze-dried to evaluate the storage stability at different temperature. It was shown that encapsulated bacteria had much better stability at 23°C for 12 weeks, and lecithin helped in obtaining higher efficiency and more stability [221].

### 3.3. The Other Drying Method Used for Microencapsulation

The other drying techniques are listed below.

#### 3.3.1. Fluidized Bed Drying

In this process, cell suspension is sprayed and dried on inert carriers using a Wurster-based fluidized bed system (Table 9) [222]. 

#### 3.3.2. Vacuum Drying

Vacuum drying is suitable for heat sensitive probiotics because drying takes place at lower temperatures, and oxidation reaction can also be minimized, while disadvantage is batch operation and longer drying time which can be minimized by using a continuous vacuum dryer where cost is one-third of a freeze dryer, and the material can be dried at 1–4% moisture level at 40°C within 5–10 min [223].

### 3.4. Another Technique for Encapsulation

#### 3.4.1. Spray Freeze Drying

In this technique, the probiotic cell solution is atomized into a cold vapor phase of a cryogenic liquid such as liquid nitrogen, which generates a dispersion of frozen droplets. These are dried in freeze dryer (Table 10) [200, 209, 210, 224]. 

#### 3.4.2. Encapsulation by Coating and Agglomeration

**Figure 8:**
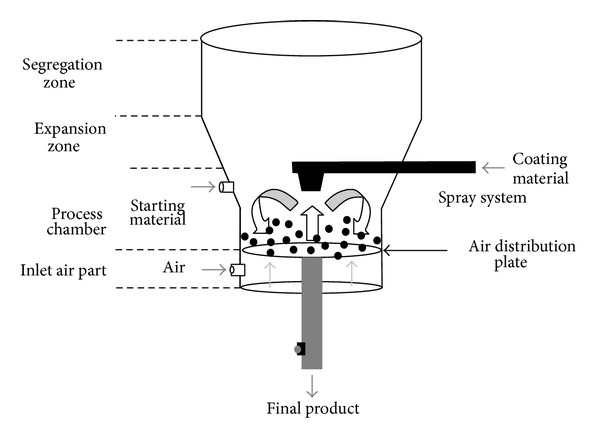
Schematic presentation of the spray coating technology.

In this method, solid form of core material is kept in motion in a specially designed vessel (Figure 8) [206, 210]. 

It is easy to scale up hence used in the encapsulation of probiotics for nutraceutical. The Canadian private company developed and patented a microencapsulation technique known as Probiocap [225]. The process is based on coating freeze-dried *Lactobacillus* with fatty acids. This technology allows strains to resist the harsh effect of temperature, gastric acidity, and compression. Danish-Korean Company patented a duel coating technology for *Lactobacillus*, which is marketed under the brand name Duaolac. The first layer of coating is made of soy peptide, and the second layer is made of cellulose and gum.

#### 3.4.3. Coacervation Technique for Encapsulation

In coacervation process, colloidal particle is separated from a solution and deposited around core material. It is used in encapsulating flavor oil but is also used in fish oil, vitamin, enzyme, nutrients, and preservatives (Table 11). It is a three-step process comprising of phase separation, deposition, and solidification [26]. In the first step, coating material containing one or more polymer goes through a phase separation process and forms a coacervate. Suspended or emulsified form of core material remains, and as soon as wall material particles coalesce, it causes a decrease in surface area and total free interfacial energy of the system. In this process, coacervate nuclei adsorption to the surface of core material and form uniform layer around the core particles. Finally solidification of coating material is done by cross-linking using chemical, thermal, or enzymatic method. The formed microparticles are then collected by filtration or mild centrifugation followed by drying [26, 193].

Glutaraldehyde, cross-linking agent, is not applied in the food industry due to toxicity issues; thus cross-linking enzyme transglutaminase is used [26, 226].

#### 3.4.4. Cocrystallization

It is mainly used for the fruit juices, essential oils, flavor, and brown sugar [193]. In this method, core material is dispersed in supersaturated sucrose solution maintained at high temperature. The heat is gradually released allowing the solution to crystallize with the core material. Finally the product is dried and sieved as per the particle size requirement (Table 12) [227]. 

#### 3.4.5. Molecular Inclusion

This method involves entrapment of smaller molecule inside the hollow cavity of a larger molecule [193, 228]. Cyclodextrins are commonly used but restricted in the certain countries. In controlled release mechanism, core material is released when displaced by more favorable substrates. It is reported that *β*-cyclodextrin molecules containing core compound are highly heat stable, can tolerate up to 200°C, and are highly resistant to chemical degradation [146, 228].

Some of the major limitations of this molecular inclusion technology are low payload [146] and high cost of raw material [193].

#### 3.4.6. Centrifugal Extrusion Technique

In this technique, core and coating materials are pumped through a separate tube to the surface of rotating cylinder. With the rotational motion of the cylinder, both materials are mixed and extruded as a fluid rod which is broken by the centrifugal force. The coating over the core material forms capsules caused by the difference in surface tension. Finally, formed capsules are placed on a moving bed of starch, which absorbs excess moisture and cushion the impact [26].

It is used in the food industry to encapsulate ingredients such as a flavor and seasoning [26], aspartame, vitamin, and methionine [19]. It produces smaller particles with a wide range of coating materials such as gelatin, alginate, carrageenan, starches, fatty acid, and waxes [19, 26].

The major advantage of this method is slower release properties of the capsule and higher throughput rate in comparison to the spray drying process [146].

## 4. Biomaterials Used for Microencapsulation of Probiotics

This section aims to provide a short overview of commonly used bilateral to encapsulate probiotic cells.

*Definition*. “Any natural material or not, which is in contact with a living structure and is intended to act with biological system.” It includes natural and synthetic polymerswhich are directly in contact with living cell so they should be biocompatible and biodegradable [229]. Encapsulation of probiotics in biodegradable polymer matrix has a number of advantages. Cryo- and osmoprotection agent can be incorporated into the matrix which enhances the survival of cell during storage and processing. Finally, microcapsules are dried; surface coating is altering the aesthetic and sensory properties of product and provides a high level of protection to the cells. It helps in the delayed release of cell by maintaining the dissolution properties of the coating layer. Microcapsule produced by using polymer is easy on a lab scale. But the scaling process is very difficult and processing cost is very high. 

### 4.1. Use of Alginate System for Encapsulation of Probiotics

Alginate is a naturally derived polysaccharide extracted from various species of algae and composed of two monosaccharide units: *α*-L-guluronic acid (G) and *β*-D-mannuronic acid (M) linked from *β* (1–4) glycosidic bond [230, 231]. M/G ratios determine the technological functionality of alginate. The gel strength depends upon high proportion block G. High temperature (60°C to 80°C) is needed to dissolve alginate in water. Alginate gels are insoluble in acidic media [6, 165, 232, 233]. Usually alginate is used in concentration range of 0.5–4% (Table 13) [27]. 

### 4.2. Use of Chitosan for Encapsulation of Probiotics

Chitosan is a linear polysaccharide with negative charge arising from its amine groups obtained by deacetylation of chitin. It can be isolated from crustacean shells, insect cuticles, and the membranes of fungi. It is a copolymer of two monomer residues anhydro-N-acetyl-D-glucosamine and anhydrous-D-glucosamine. It is soluble at pH < 6 and forms gel structure by ionotropic gelation. Chitosan can further polymerize by means of cross-linking formation in the presence of anions and polyanions [202]. It is used for coating of gelatin capsules, because its efficiency for the increasing viability of probiotic cells is not satisfactory; it is most often used as coat/shell but not capsule. 

### 4.3. Use of Starch for Encapsulation of Probiotics

Starch consists of D-glucose unit joint together with glycosidic bonds. It has been used as a material for coating of alginate capsules. High-amylose corn starch (HACS) can be applied for enhancing functions of capsule or shell/coat formation [183]. Lyophilized corn starch (LCS) has been reported to be used as capsule-forming material; however, it decomposes after being subjected to pancreatic enzymes [234]. Resistant starch (RS) is not degraded by the pancreatic amylase. His specification apart from giving the microbeads good enteric delivery characteristic also gives them probiotic functionality as they can be used by the probiotic bacteria in the intestine [235]. The incorporation of Hi-Maize starch improved the encapsulation of viable bacteria compared with the bacteria encapsulated without starch [140, 236]. 

### 4.4. Use of Xanthan-Gellan Gum for Encapsulation of Probiotics

Gellan gum is an anionic polysaccharide derived from *Sphingomonas elodea* which is constituted of a repeating unit of four monomers that are glucose, glucuronic acid, glucose, and rhamnose [22]. Xanthan is an exopolysaccharide derived from *Xanthomonas campestris*. The optimum mixing proportion for xanthan-gellan gum is 1 : 0.75 [32]. In contrary with alginate, this mixture is resistant to acidic conditions [32, 181]. 

### 4.5. Use of *κ*-Carrageenan for Encapsulation of Probiotics

Carrageenan is polymer having linear structure consisting of D-galactose units alternatively linked by *α*-(1–3) and *β* (1–4) bonds. Types of carrageenan are kappa (*κ*), iota (*ι*), and lambda (*λ*) [237]. Monosulfated *κ*-carrageenan and bisulfated *ι*-carrageenan contain oxygen bridge between 3 and 6 of the D-galactose, which is responsible for the conformational transition and gelatin. The *λ*-carrageenan is trisulfated and does not have this bridge required for gel formation [238]. Carrageenan gelatin is induced by temperature changes. A rise in temperature (60–80°C) is required to dissolve it, and gelation occurs by cooling to room temperature [238, 239], and then microparticles are stabilized by adding potassium ion [29]. It is commonly used as a food additive; its safety has been approved by several government agencies including FDA, Codex Alimentarius, and the joint FAO/WHO food additive [240]. It has good capacity to form gels that can entrap the cell. However, the cell slurry should be added to the heat sterilized suspension between 40–45°C; otherwise the gel hardens at room temperature [241]. Usually it is used in concentration such as 2–5% [242]. The encapsulation of probiotic cell in *κ*-carrageenan beads keeps the bacteria in a viable state [169], but the produced gels are brittle and do not withstand stresses [22].

### 4.6. Use of Various Proteins-Based Coating for Encapsulation of Probiotics

#### 4.6.1. Gelatin

Gelatin is used as a thermally reversible gelling agent for encapsulation. Because of its amphoteric nature, it is an excellent candidate to incorporate with anionic-gel-forming polysaccharides, such as gellan gum.

It is frequently used in food and pharmaceutical industries [243]. It is a protein derived by partial hydrolysis of collagen of animal origin. It has versatile functional properties, and forms a solution of high viscosity in water which set to a gel on cooling.

#### 4.6.2. Milk Protein

Milk proteins are natural vehicles for probiotic cells, and owing to their structural and physicochemical properties, they can be used as a delivery system [217]. The results of these studies are promising, and using milk proteins is an interesting way because of their biocompatibility [217].

#### 4.6.3. Whey Protein

It easily heats denatured which affect aggregation and reduction in emulsion stability. Whey proteins are heat sensitive and show inferior surface activities. Whey protein appears as a potential candidate as coating agent as it is entirely biodegradable and frequently used in many types of food products.

The protein matrixes have different cell release properties than the other microencapsulation methods (polymer or fat based). Thus, applications are also extended to other foods for protection during processing as well as stability during storage but also in nutraceutical for protection and soil release in the GI tract [244].

### 4.7. Use of Cellulose Acetate Phthalate (CAP) for Encapsulation of Probiotics

Because of its safe nature, CAP is used for controlling drug release in the intestine [23]. It is not soluble at pH less than 5 but it is soluble at pH higher than 6 [236]. This property is essential for probiotic encapsulation because the bilateral must not dissolve in the stomach but only in the gut. The disadvantage of CAP is that it cannot form gel beads by ionotropic gelation so capsules have been developed by emulsification. CAP is widely used as a coating agent because it provides better protection for microorganisms in simulated GI conditions [191].

### 4.8. Criteria to Select a Proper Encapsulation Technology

When one chooses encapsulation as a technology to deliver the desired benefits, one should consider carefully the design of the encapsulation.

- What are the physicochemical characteristics of the active?
- Which processing conditions are used during food production or processing?
- How will the encapsulates be stored prior to use?
- What will be the storage conditions of the food product containing the encapsulates prior to consumer use?
- Which particle size and density are needed to have it incorporated properly in the food product?
- What are the trigger(s) and mechanism(s) of release?
- What are the cost constraints?

## 5. Conclusion and Future Perspective

In the present article, principle, methods, and materials used in the encapsulation of probiotic cells are discussed. The advances in this field have been tremendous with nutraceutical and food ingredients. However, as to the microencapsulation of live probiotic bacterial cells, the technology seems to be not well developed. The delivery of viable microencapsulated probiotic bacteria will become important in near future. Any type of triggers can be used to prompt the release of encapsulated ingredients, such as pH changes, mechanical stress, temperature, enzymatic activity, time, and osmotic force. The challenges are to select the appropriate encapsulation technique and encapsulating materials. One important challenge for cell encapsulation is the largest size of microbial cells (typically 1–4 *μ*m) or particles of freeze-dried culture (more than 100 mm). This characteristic limits cell loading for small capsules or, when large size capsules are produced, can negatively affect the textural and sensorial properties of food products in which they are added. In almost all cases, gel entrapment using natural polymers, such as calcium alginate, carrageenan, gellan gum, and Chitosan, is favored by researchers. However, despite promising on a laboratory scale, the developed technologies for producing gel beads still present serious difficulties for large-scale production of food grade microencapsulated microorganisms.

Another major challenge is to improve the viability of probiotics during the manufacturing processes, particularly heat processing. Consequently, there appears to be no commercial probiotic products available that are stable at high temperatures. Keeping in view the importance of producing thermoresistant probiotic microorganisms, as well as the interests of food and pharmaceutical companies, new approaches are needed in further research. There are at least two options: (1) discovering new strains of probiotic bacteria that are naturally heat stable or that have been genetically modified and (2) developing an encapsulation system that effectively acts like an “insulation material.”

## Figures and Tables

**Table 1:** Clinical studies of appropriate probiotic strains which have convincingly demonstrated their therapeutic effect.

Most appropriate probiotic strain(s)	Therapeutic application	References
*Lactobacillus plantarum *299v*, Bacillus coagulans *ATCC no. 31284, and *Lactobacillus acidophilus *L1	Hypercholesterolemia and cardiovascular disease	[33–36]
*Lactobacillus rhamnosus *GG	Prevention of atopy	[37, 38]
*Lactobacillus rhamnosus *GG, *Bifidobacterium lactis*, and *Lactobacillus paracasei *	Eczema	[39–44]
*Lactobacillus rhamnosus *GG, *Bifidobacterium lactis*, and *Lactobacillus paracasei *	Food allergies	[40–49]
*Lactobacillus rhamnosus*, *Bifidobacterium lactis*, *Lactobacillus johnsonii, Lactobacillus rhamnosus*, and; *Lactobacillus acidophilus *	Lowered immunity	[50–60]
*Lactobacillus rhamnosus *GG, *Saccharomyces cerevisiae*, *Lactobacillus acidophilus,* and *Lactobacillus plantarum *299v	Antibiotic use (during and after)	[61–72]
*Lactobacillus rhamnosus *GG	Nonsteroidal anti-inflammatory Drug	[73]
*Lactobacillus rhamnosus *GG, *Saccharomyces cerevisiae *	Intestinal hyperpermeability	[41, 42, 74, 75]
*Lactobacillus rhamnosus *GG, *Lactobacillus reuteri *MM53, *Lactobacillus paracasei *CRL431, *Lactobacillus acidophilus *CRL730, *Lactobacillus johnsonii *La1, *Bifidobacterium lactis *Bb12, *Lactobacillus plantarum *299v, and *Lactobacillus paracasei *	Gastroenteritis	[40, 41, 76–85]
*Lactobacillus johnsonii *La1^*α*^, *Lactobacillus plantarum *299v*, *and *Lactobacillus rhamnosus *GG	Giardia infection	[77, 86, 87]
*Lactobacillus rhamnosus *GG, *Lactobacillus johnsonii *La1, *Lactobacillus plantarum *299v, *Lactobacillus paracasei*, and *propionibacterium freudenreichii* HA-101 and HA-102	Intestinal dysbiosis	[64, 65, 88–93]
*Lactobacillus acidophilus, Lactobacillus johnsonii *La1	Lactose intolerance	[94–98]
*Lactobacillus johnsonii *La1, *Lactobacillus acidophilus*, and *Lactobacillus rhamnosus *GG	Peptic ulcer disease Nonerosive gastritis	[50, 99–102]
*Lactobacillus plantarum *299v, VSL no. 3^*β*^	Irritable bowel syndrome	[103–105]
*Lactobacillus acidophilus *NCFB 1748, VSL no. 3^*α*^	Radiation-induced diarrhoea	[106, 107]
*Lactobacillus rhamnosus *GG, *Bifidobacterium lactis *Bb12, *Lactobacillus acidophilus*, *Saccharomyces cerevisiae*, and *Lactobacillus plantarum *299v	Traveller's diarrhoea	[77, 84, 108–110]
*Lactobacillus rhamnosus *GG, *Saccharomyces cerevisiae *	Crohn's disease	[111–114]
*Escherichia coli *Nissle 1917, VSL no. 3^*β*^, *Lactobacillus plantarum *299	Ulcerative colitis	[115–119]
*Lactobacillus rhamnosus *GG, *Lactobacillus acidophilus*, *Lactobacillus paracasei*, *Lactobacillus acidophilus, *and *Lactobacillus delbrueckii *ssp. *bulgaricus *strain LB-51	Prevention of colon cancer	[120–124]
*Lactobacillus rhamnosus *GR-1*, Lactobacillus fermentum *B-54, *Lactobacillus fermentum *RC-14, and *Lactobacillus acidophilus *	Urinary tract infection	[125–132]
*Lactobacillus acidophilus*, *Lactobacillus rhamnosus *GG, *Lactobacillus rhamnosus *GR-1, and *Lactobacillus fermentum *RC-14	Vaginal candidiasis (thrush)	[133–138]

**Table 2:** Supported report provides evidence that encapsulation of probiotics results in increased viability.

Sr. no.	Supported report provides evidence that encapsulation of probiotics results in increased viability	References
1	It has been investigated that when yoghurt isolates of *L. acidophilus* and *Bifidobacterium* are encapsulated in 2% alginate solution, the viability was increased 15.9% and 16.6%, respectively, under acidic and bile salt condition	[139]
2	The use of calcium-induced alginate starch coating has also improved the survivability of encapsulated cells of *Lactobacillus acidophilus* and *Bifidobacterium lactis* (probiotic bacteria) up to 2 and 1 log cell numbers, respectively, in yoghurt	[140]
3	Whey protein-based microcapsules can improve the cell survival of probiotic bacteria under extreme conditions	[141]
4	A combination of gellan-alginate was used to encapsulate *Bifidobacterium bifidum*. The result showed that 2% sodium alginate and 1% gellan gum used as encapsulating materials have provided the highest thermotolerance in terms of *B. bifidum* count. The results of heat treatments also demonstrated that the addition of gellan gum in the walls of probiotic microcapsules provided improved protection for *B. bifidum*. The cell counts of *B. bifidum* remained at 10^5^–10^6^ CFU/g for the microcapsules stored for 2 months	[142]
5	Encapsulated cells also showed approximately 10^4^ times increase in viability during exposure to acidic and bile salt conditions	[143]
6	It was found that cells microencapsulated in alginate, carrageenan, and xanthan gum survived better than free cells following 2 h incubation in acidic condition (pH 2)	[144]
7	It was found that cells encapsulated by extrusion using alginate and alginate with starch offered greater protection to cells in simulated gastric juice	[24]
8	Chitosan coating of microbeads resulted in a significant increase in survival time of *L. rhamnosus* from 40 to 120 min in acid condition, and the reduction in cell numbers was confined to 0.94 log over this time. Alginate macrobeads are more effective than microbeads in protecting *L. acidophilus* against high acid and bile	[145]

**Table 3:** Relative pH and transit time at various locations within GIT.

Region	pH	Transit time
Oesophagus	~7.0	10–14 seconds
Stomach	1–2.5 (up to 5 fed)	Half emptying: ~80.5 mins
Proximal small intestine	6.15–7.35	3.2 ± 1.6 hrs (combined)
Distal small intestine	6.80–7.88	
Ascending colon	5.26–6.72	Highly variable, dependent on bowel evacuation
Descending colon	5.20–7.02	

**Table 4:** Advantage and disadvantages of extrusion technique.

Advantages	Disadvantages
(i) Simple and cheap method that uses a gentle operation (ii) No damage to the probiotic cell (iii) Gives high probiotic viability [29] (iv) Does not involve deleterious solvent (v) Done under aerobic and anaerobic conditions	(i) Difficult to use for large scale production due to slow formation of microbeads (ii) Very poor payload of typically 8% (iii) Susceptibility of carbohydrate towards damage and structural defect, a larger size distribution (iv) Limited choice of wall material [146, 147].

**Table 5:** Different probiotic strain, biomaterial, and size of microcapsule encapsulated by extrusion technique.

Probiotic strain	Material	Size of capsule	Reference
*Lactobacillus bulgaricus*, *Streptococcus thermophilus *	1.875% alginate	2.5 mm	[148]
*Streptococcus lactis* ssp.* diacetylactis* *Streptococcus cremoris *	1.875% alginate	2.6 mm	[149]
*Streptococcus cremoris *	1% alginate	—	[150]
*Lactococcus lactis* ssp.* cremoris *	2% alginate + 0.4% chitosan	2 mm	[12, 147]
*Lactobacillus plantarum *	2% Alginate + 10% skim milk	2 mm	[151]
*Lactococcus lactis* ssp.* lactis bv. diacetylactis *	1.5% alginate	—	[152]
*Lactococcus lactis* ssp.* cremoris *	2% alginate	—	[153]
*Streptococcus thermophilus* *Lactobacillus delbrueckii* ssp. *bulgaricus *	2% alginate	—	[154]
*Lactococcus lactis* ssp.* lactis bv. diacetylactis *	1.8% alginate		[155]
*Lactobacillus acidophilus *	0.6% alginate + starch	5 mm	[156]
*Bifidobacterium lactis *	0.75% gellan gum + 1% xanthan gum	3 mm	[32, 157, 158]
*Lactobacillus reuteri *	1% xanthan gum + 0.5% gellan gum	—	[24]
*Lactococcus lactis *	1% alginate, poly-L-lysine	2 mm	[159]
*Lactobacillus reuteri *	1.5% alginate + 0.1% poly-L-lysine and 0.1% alginate	619 *μ*m	[160]
*Lactobacillus *spp.	1.8% alginate	330–450 *μ*m	[161]
*Lactococcus lactis* ssp.* cremoris *	2% alginate	1.62 mm	[12]
*Lactococcus lactis* ssp.* cremoris *	2% alginate + 0.17% alginate	1.89 mm	[12]
*Saccharomyces boulardii *	1.8% alginate + 0.4% chitosan	356 *μ*m	[162]
*Lactobacillus reuteri *	2% alginate + 2% corn starch	—	[24]
*Lactococcus lactis* ssp.* cremoris *	2% alginate + 0.05% poly-L-lysine	1.89 mm	[12]
*Bifidobacterium bifidum *	2% alginate, 1% gellan, 0.86% peptides, 0.2% FOS	—	[163]
*Lactobacillus acidophilus, Lactobacillus casei, Bifidobacterium bifidum,* and *Bifidobacterium longum *	3% alginate, 1% peptides, 3% FOS	—	[164]
*Bifidobacterium longum *	2–4% alginate	1.03–2.62 mm	[165]
*Lactobacillus reuteri, Escherichia coli *	3% alginate	—	[24, 166, 167]
*Lactobacillus reuteri *	1.75% *κ*-carrageenan + 0.75% locust bean gum	—	[24]
*Lactobacillus acidophilus, Bifidobacterium lactis *	1.8% alginate + 1% Hi-Maize starch	—	[140, 168]
*Bifidobacterium bifidum *	3% *κ*-carrageenan	—	[169]

**Table 6:** Different probiotic strain, biomaterial, and size of microcapsule encapsulated by emulsion technique.

Probiotic strain	Material	Size of microcapsule	Reference
*Streptococcus thermophilus *	3% *κ*-carrageenan and locust bean gum (2 : 1)	0.5–2 mm	[170]
*Bifidobacterium pseudolongum *	10% cellulose acetate phthalate	—	[171]
*Lactobacillus delbrueckii* ssp.* bulgaricus *	3% alginate	—	[172]
*Lactobacillus casei* ssp.* casei *	3% *κ*-carrageenan and locust bean gum (11 : 1)	1-2 mm	[173]
*Lactococcus lactis* ssp. *cremoris *	Chitosan (4%)	150 *µ*m	[174]
*Lactobacillus delbrueckii* ssp.* bulgaricus *	3.6% alginate	30 *µ*m	[175]
*Lactobacillus delbrueckii* ssp.* bulgaricus *	3% alginate	25–35 *µ*m	[27]
*Lactococcus lactis* ssp. *cremoris *	24% gelatin	271–168 *µ*m	[176]
*Lactococcus lactis* ssp. *cremoris *	2% alginate	50 *µ*m–1 mm	[177]
*Bifidobacterium bifidum, Bifidobacterium infantis *	3% alginate	—	[28]
*Lactobacillus casei *NCDC-298	2–4% alginate	—	[178]
*Lactobacillus bulgaricus *KFRI 673	2% alginate, 5% glycerol, 0.26% xanthan gum + 0.8% chitosan	40–80 *µ*m	[179]
*Lactobacillus reuteri *	2% alginate + 2% corn starch	—	[24]
*Lactobacillus acidophilus, Bifidobacterium lactis *	2% alginate + 2% Hi-Maize starch	0.5–1 mm	[140, 180, 181]
*Lactobacillus reuteri *	1.75% *κ*-carrageenan + 0.75% locust bean gum	—	[24]
*Bifidobacterium breve *	Milk fat + 10% whey protein isolate	3–80 *µ*m	[141]
*Lactobacillus reuteri *	1% xanthan + 0.5% gellan		[24]
*Bifidobacterium adolescentis* 15703T	13% gelatin, 1.25 mM genipin + 1% alginate	49–53 *µ*m	[182]

**Table 7:** Different probiotic strain, biomaterial, inlet/outlet temperature, and size of microcapsule encapsulated by spray drying techniques.

Probiotic strain	Material	Inlet/outlet temperature	Size of capsule *μ*m	Reference
*Bifidobacterium infantis* CCRC 14633, *Bifidobacterium infantis* CCRC 14661, *Bifidobacterium longum* ATCC 15708, *Bifidobacterium longum* CCRC 14634, and* Bifidobacterium longum* B6	10% gelatin, gum arabic, soluble starch, or skim milk	100°C/50°C	10–20	[183–185]
*Lactobacillus paracasei* NFBC 338	20% skim milk	175°C/68°C	—	[186]
*Lactobacillus rhamnosus* GG	20% skim milk + Raftilose or Polydextrose	—/80°C		[187]
*Bifidobacterium breve* R070 (BB R070), *Bifidobacterium longum* R023 (BL R023), and* Lactobacillus acidophilus* R335 (LA R335)	10% whey protein isolate	160°C/80°C	5–80	[188]
*Bifidobacterium* PL1	10% waxy maize starch	100°C/45°C	5	[189]
*Lactobacillus acidophilus* BCRC 14079*, Bifidobacterium longum* BCRC 14605	30% maltodextrin + 20% gum arabic	100°C/50°C	10	[190]
*Lactobacillus acidophilus* La-05 *Bifidobacterium lactis* Bb-12	Cellulose acetate phthalate	130°C/75°C	22	[191]
*Lactobacillus paracasei* NFBC 338	Gum acacia (gum arabic)	170°C/95–105°C	5–15	[192]

**Table 8:** Advantage and disadvantages of spray drying techniques.

Advantages	Disadvantages
The advantages of the spray drying process are ease of scaling up, low operational cost, continuous operation, and adaptability to most common industrial equipment	(i) However, spray drying may not be suitable particularly for probiotic bacteria due to requirement of high temperature drying [19, 193, 194] (ii) The loss of viability also depends upon the type of carrier used, for example, log reduction in soluble starch found to be higher compared to other carriers such as alginate, gum Arabic, and skim milk [183, 195]

**Table 9:** Advantage and disadvantages of fluidized bed drying.

Advantages	Disadvantages
The advantages of this process are total control over the temperature, lower comparable cost	Disadvantage being relatively longer duration (up to 2 hours) [195]. Before this drying, probiotic culture is encapsulated first in supporting material such as skimmed milk [196], potato starch [197], calcium alginate [198], or casein [199]

**Table 10:** Advantages and Disadvantages of spray freeze drying.

Advantages	Disadvantages
The advantages are controlled size, large specific surface area than spray dried capsule	(i) Disadvantages are required high energy, long time, expensive than spray drying [21] (ii) Additional coating is given to capsule for protection against adverse environmental conditions [200]

**Table 11:** Advantages and disadvantages of coacervation technique.

Advantages	Disadvantages
(i) The advantages are high payload (99%) and controls the release of core material [146]	(i) The disadvantages are high cost of the particle isolation procedure and complexity of technique
(ii) The process can be carried out at room temperature making it particularly suitable for heat sensitive probiotic bacteria [26]	(ii) But it was suggested that the optimization of the last step and use of spray dryer instead of fluidized or freeze dryer can reduce the overall cost [146]

**Table 12:** Advantages and disadvantages of cocrystallization.

Advantages	Disadvantages
The advantages are economical with high payload (90%). It is utilized in the confectionery and pharmaceutical industries	Disadvantages are higher control rate of nucleation, crystallization, and thermal balance during operation [26]

**Table 13:** Advantages and disadvantages of alginate system.

Advantages	Disadvantages
(i) The advantages are easy formation of gel matrices around bacterial cells, being nontoxic and cheap; mild process conditions are needed for their performance, easily prepared and performed and properly resolved in the intestine and release entrapped cell [85, 146, 201] (ii) Alginate gel matrix forms bacterial cell beads with a diameter of 1–3 *µ*m, and the pore size does not exceed 7 NM [202]	(i) Disadvantages are susceptible to acidic environments, crackling and loss of mechanical stability in lactic acid [203, 204], difficulties in industrial scale applications due to their high expenses, and a weak ability of scaling up as well as the formation of crackled and porous bead surfaces, which leads to fast diffusion of moisture and fluids through capsules which reduces their barrier properties against unfavorable environment factor [146] (ii) This can be overcome by blending of alginate with another polymer like starch, coating other compound on its capsules [29, 30, 32, 181]
